# Dementia incidence trend over 1992-2014 in the Netherlands: Analysis of primary care data

**DOI:** 10.1371/journal.pmed.1002235

**Published:** 2017-03-07

**Authors:** Emma F. van Bussel, Edo Richard, Derk L. Arts, Astrid C. J. Nooyens, Preciosa M. Coloma, Margot W. M. de Waal, Marjan van den Akker, Marion C. J. Biermans, Markus M. J. Nielen, Kees van Boven, Hugo Smeets, Fiona E. Matthews, Carol Brayne, Wim B. Busschers, Willem A. van Gool, Eric P. Moll van Charante

**Affiliations:** 1 Department of General Practice, Academic Medical Center Amsterdam, Amsterdam, The Netherlands; 2 Department of Neurology, Donders Institute for Brain, Cognition and Behavior, Radboud University Medical Center, Nijmegen, The Netherlands; 3 Department of Neurology, Academic Medical Center Amsterdam, Amsterdam, The Netherlands; 4 Centre for Nutrition, Prevention and Health Services, National Institute for Public Health and the Environment, Bilthoven, The Netherlands; 5 Department of Medical Informatics, Erasmus University Medical Center, Rotterdam, The Netherlands; 6 Department of Public Health and Primary Care, Leiden University Medical Center, Leiden, The Netherlands; 7 CAPHRI School for Public Health and Primary Care, Department of Family Medicine, Maastricht University, Maastricht, The Netherlands; 8 Academic Center for General Practice, Department of Public Health and Primary Care, KU Leuven, Leuven, Belgium; 9 Department of Primary and Community Care, Radboud University Medical Center, Nijmegen, The Netherlands; 10 Department of General Practice, Netherlands Institute for Health Services Research, Utrecht, The Netherlands; 11 Julius Center for Health Sciences and Primary Care, University Medical Center Utrecht, Utrecht, The Netherlands; 12 Institute for Health and Society, Newcastle University, Newcastle upon Tyne, United Kingdom; 13 Institute of Public Health, Cambridge University, Cambridge, United Kingdom; University of California San Francisco Memory and Aging Center, UNITED STATES

## Abstract

**Background:**

Recent reports have suggested declining age-specific incidence rates of dementia in high-income countries over time. Improved education and cardiovascular health in early age have been suggested to be bringing about this effect. The aim of this study was to estimate the age-specific dementia incidence trend in primary care records from a large population in the Netherlands.

**Methods and findings:**

A dynamic cohort representative of the Dutch population was composed using primary care records from general practice registration networks (GPRNs) across the country. Data regarding dementia incidence were obtained using general-practitioner-recorded diagnosis of dementia within the electronic health records. Age-specific dementia incidence rates were calculated for all persons aged 60 y and over; negative binomial regression analysis was used to estimate the time trend. Nine out of eleven GPRNs provided data on more than 800,000 older people for the years 1992 to 2014, corresponding to over 4 million person-years and 23,186 incident dementia cases. The annual growth in dementia incidence rate was estimated to be 2.1% (95% CI 0.5% to 3.8%), and incidence rates were 1.08 (95% CI 1.04 to 1.13) times higher for women compared to men. Despite their relatively low numbers of person-years, the highest age groups contributed most to the increasing trend. There was no significant overall change in incidence rates since the start of a national dementia program in 2003 (−0.025; 95% CI −0.062 to 0.011). Increased awareness of dementia by patients and doctors in more recent years may have influenced dementia diagnosis by general practitioners in electronic health records, and needs to be taken into account when interpreting the data.

**Conclusions:**

Within the clinical records of a large, representative sample of the Dutch population, we found no evidence for a declining incidence trend of dementia in the Netherlands. This could indicate true stability in incidence rates, or a balance between increased detection and a true reduction. Irrespective of the exact rates and mechanisms underlying these findings, they illustrate that the burden of work for physicians and nurses in general practice associated with newly diagnosed dementia has not been subject to substantial change in the past two decades. Hence, with the ageing of Western societies, we still need to anticipate a dramatic absolute increase in dementia occurrence over the years to come.

## Abstract 2

Using primary care data, Emma van Bussel and colleagues examine trends in dementia incidence trend over 1992-2014 in the Netherlands.

## Author summary

**Why was this study done?:**

- The absolute number of persons with dementia is rising due to a growing and ageing population.
- Recent studies showed a decline in dementia incidence rates that might be attributed to improved vascular care and better education in more recent years.
- Available studies were based on relatively small samples collected in specific years. Large-scale data with yearly incidence rates were so far lacking.

**What did the researchers do and find?:**

- In the Netherlands, all data on dementia diagnoses from general practitioner networks between 1992 and 2014 were collected, yielding over 23,000 incident dementia cases in 4 million person-years.
- We found that the incidence of registered dementia cases has slightly increased during this 23-year period.

**What do these findings mean?:**

- Based on these registry data, the age-specific incidence of dementia has not declined over the last two decades.
- Increased awareness of the disease may have led to earlier diagnosis, which could have influenced the number of registered dementia cases.

## Introduction

Since dementia care places a heavy social and economic burden on society, future projections of dementia prevalence rates are important for health care planning. In view of a growing and ageing population, an increasing number of older people are at risk for dementia [1]. It is estimated that the prevalence of dementia will nearly double every 20 y, to 132 million in 2050 worldwide [2]. Recently, cohort studies from Europe and the United States have suggested a declining trend in age-specific dementia incidence rates over the last 30 y [3–12]. This putative decline is mostly attributed to better education and vascular risk factor treatment [5,9], and fuels hope that the absolute increase in dementia prevalence might be more moderate than previously anticipated.

Until recently, studies on trends in dementia occurrence have been surprisingly rare. European studies that attempted to quantify changes in incidence or prevalence over time often suffered from decreasing response rates and changing methods for dementia case identification between time points [13]. Furthermore, most studies were based on local or regional data using population-based research cohorts, rather than on nationwide registries within real-world settings [4,13].

Data from electronic health records (EHRs) may facilitate time-trend analyses, provided that the populations studied are representative and that diagnostic algorithms and procedures are relatively stable over time. In the Netherlands, nearly all non-institutionalized inhabitants are registered with a single general practitioner (GP), and morbidity is recorded through EHRs [14]. In 1988, the first national dementia guideline appeared, followed by a primary care guideline 10 y later, both of which have been amended since. The diagnostic criteria for most dementia types (e.g., Alzheimer disease and vascular dementia) have not substantially changed over the last decades, but there have been increases in awareness and attention to dementia in the population. The aim of this study was to estimate age-specific dementia incidence rates and dementia incidence trends among community-dwelling older people (≥60 y) in the Netherlands over the last decades, based on GP registry data.

## Methods

### Ethics statement

In the Netherlands, no approval from an ethical committee or individual participant consent is necessary for analyzing anonymized data from general practitioner registration networks (GPRNs).

### Database characteristics and selection

In the Netherlands, routinely collected data from GPRNs are often used to monitor the incidence and prevalence of diseases in the general population [14]. GPs use the International Classification of Primary Care (ICPC) to code all diagnoses in the patient’s EHR [15], including those made by specialists after referral [16]. GPRNs collect and manage the EHR data of large numbers of associated general practices. Most operate regionally, some nationally. For this study, all eleven GPRNs that routinely and continuously collected data on morbidity and mortality in the Netherlands over the last decades were invited to participate. We aimed to include as many consecutive years per GPRN as possible. Databases of GPRNs were considered eligible if data were available for at least 5 y and registration or extraction methods had not substantially changed over time (Table 1).

**Table 1:** Characteristics of the Dutch general practice registration networks included in this study.

GPRN abbreviation	GPRN name; description/region	Time period	Population	Number of general practices or health care centers
NIVEL-PCD	Netherlands Institute for Health Services Research Primary Care Database; nationwide	2002–2013	394,360	400
IPCI	Integrated Primary Care Information; nationwide	2003–2014	315,311	307
JHN	Julius General Practitioners Network; GPs associated with Utrecht university	2000–2013	34,949	25
RNH	Registration Network Family Practices; Limburg	1991–2011	23,208	22
SMILE	Study of Medical Information and Lifestyles in Eindhoven; Eindhoven	2004–2012	13,855	9
RNUH-LEO	Registration Network University Practices Leiden and Environment; GPs associated with Leiden University	1999–2013	10,143	4
HAG	HAGnet AMC; GPs associated with the Academic Medical Center Amsterdam	1999–2013	8,184	6
CMR	Continuous Morbidity Registration Nijmegen; GPs associated with Nijmegen University	1986–2011	3,072	4
Trans	Transitieproject; regional network of GPs in Amstelveen and Friesland	1995–2008	2,969	5
Total		1986–2014	806,051	782

Population is defined as total (alive, registered) older population (age ≥ 60 y) of the GPRN in the most recent year of provided data.

GP, general practitioner; GPRN, general practitioner registration network.

### Data extraction

For all databases, count data of incident cases and person-years at risk per year, sex, and age group were directly obtained or calculated from the anonymized data. For each calendar year, data on all people aged 60 y and over were used. Dementia was defined as P70 (senile dementia/Alzheimer disease), the only code within the ICPC for dementia. Another code slightly related is P20 (memory/concentration/orientation impairment), but it is nonspecific and was disregarded. The numerator was defined as all new dementia cases (the first date the ICPC code P70 had been recorded in a patient’s EHR) per year. For the denominator, for each calendar year the number of registered person-years was calculated (NIVEL-PCD, IPCI, RNUH-LEO, CMR) or, if person-years could not be calculated, the number of registered persons (JHN, RNH, SMILE, HAG, Trans). At the start of each calendar year, prevalent dementia cases were excluded from both the numerator and denominator.

Methods of data collection are episode-based in some registries and problem-based in others. Problem-based data (“problem list”) contain information about health problems that are permanent, chronic (duration longer than 6 mo), or recurrent. Thus, for dementia, recording on the problem list is clearly expected. Episode-based data (“episode list”) have information about all health problems. In two databases, new dementia cases were identified when the ICPC code P70 was entered on the problem list (RNUH-LEO and RNH). In the seven other databases, new dementia cases were identified when the ICPC code P70 was recorded in the episode list for a patient contact [16]. Within each database, coding and selection criteria were stable over the whole time frame of the study. Although differences in data recording and data selection may cause variation in incidence or prevalence rates between GPRNs, they do not impact trends or variation within GPRNs [14].

### Statistical analysis

Data for all available years with at least 10,000 observed person-years were used for data analysis, in order to avoid unrepresentative sample years, resulting in data for the years 1992 through 2014.

To model the observed rates over time, a prespecified analysis plan was followed, and both linear and nonlinear relations were considered. Fitting count models using splines with varying degrees of freedom for the continuous variables did not reveal significant departure from linearity. Regarding the linear structures and using the log link function for the mean, both the Poisson and the negative binomial distribution were considered, the latter being able to account for overdispersed count data. Based on Akaike’s Information Criterion, it was concluded that the negative binomial distribution fit our data best. During the study, GPRNs appeared to differ individually in terms of methods of data collection and denominator calculation; therefore, we incorporated a GPRN random intercept and slope term into the negative binomial regression models to allow for GPRN-specific trends of dementia over time. The most parsimonious random structure was chosen based on the likelihood ratio test (model 1).

The time—rate relation was adjusted for age (in 5-y age groups) and sex. We also investigated whether the time—rate relation differed across age groups and sex by adding the appropriate interaction terms to the model (models 3 and 4).

Furthermore, to test the hypothesis that the recorded dementia incidence increased as a result of increased awareness and case-manager-led integrated dementia care, a piecewise linear spline was included in the model, with an internal knot at year 2003 (when a national dementia care program was launched in the Netherlands) (model 2) [17]. Additional sensitivity analyses were performed including all available data (1986–2014) in the negative binomial regression (model 5) and using Poisson regression instead of negative binomial regression (model 6).

Database preparation was performed in IBM SPSS Statistics 22; statistical analyses were performed in R version 3.1.2 using packages plyr, R2admb, and glmmADMB [18–21]. The programming codes can be requested from the corresponding author.

## Results

All eleven Dutch GPRNs were willing to participate. However, one GPRN (in Amsterdam) was able to deliver coherent incidence data only for the years 2010 to 2013 and was excluded from participation. Another GPRN (in Groningen) was excluded because its data were already part of another database within this study (NIVEL-PCD). The other nine databases, representing over 806,051 older persons, were eligible and were used for this trend study (Table 1).

Registration periods were between 1986 and 2014 and ranged from 9 to 26 y across the networks. Populations covered by the individual GPRNs ranged from 2,969 to 394,360 older people, with the two largest networks operating nationally. From 1992 onwards, at least 10,000 person-years were available for each year; thus, the years 1992–2014 were used for data analysis.

**Fig 1:**
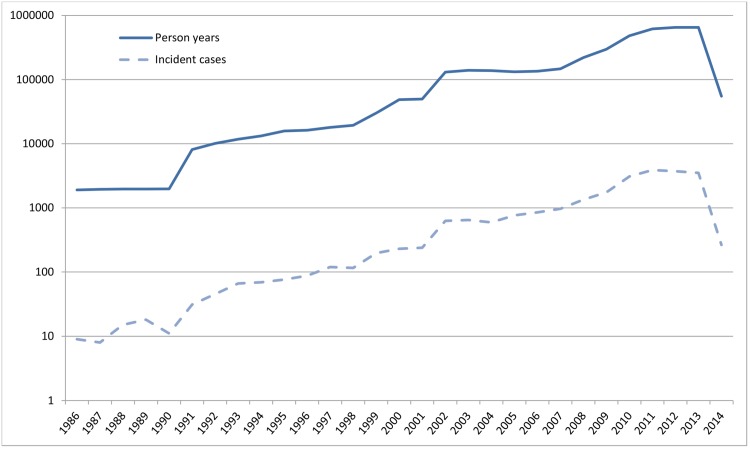
Absolute number of person-years at risk and incident dementia cases per calendar year (logarithmic *y*-axis).

Fig 1 shows the number of person-years at risk and the number of incident dementia cases for each calendar year. Between 1992 and 2014, a total of 4,020,550 person-years were available, during which 23,186 incident cases of dementia were recorded. Table 2 shows the crude mean incidence rate per age group and its range across GPRNs. The incidence of dementia increased with age in all of the individual databases.

**Table 2:** Crude mean and range of dementia incidence rate for all nine general practitioner registration networks (1992–2014), and number and percentage of person-years per age group.

Age group (years)	Number of person-years	Percent of total person-years	Crude mean incidence rate (per 1,000 person-years)	Range of mean incident rate across GPRNs
60–64	1,126,891	27.5	0.44	0.21–0.65
65–69	919,145	23.6	1.13	0.75–1.77
70–74	711,079	18.8	3.19	1.20–4.74
75–79	554,675	14.1	8.18	3.73–15.08
80–84	389,241	9.3	16.35	8.91–30.00
85+	319,521	6.7	26.53	12.38–47.08
Total	4,020,550	100	5.77	2.70–8.69

GPRN, general practitioner registration network.

**Fig 2:**
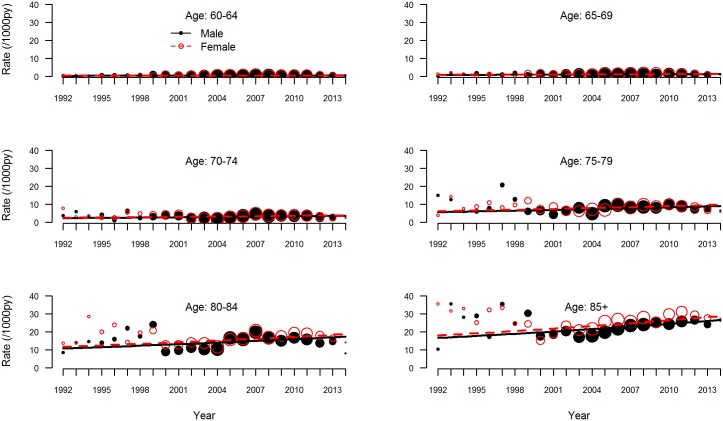
Dementia incidence rate by age group. Observed (circles) and estimated (lines) dementia incidence rate per 1,000 person-years (py) by age group for men (solid black circles and lines) and women (open red circles, dashed lines). The sizes of the circles indicate the number of general practitioner registration networks that provided data for the respective years.

The observed and estimated trend of the incidence rate per age group are shown in Fig 2. The dementia incidence rate ratio was 1.021 (95% CI 1.005 to 1.038), reflecting an annual growth in dementia incidence rate of 2.1% (95% CI 0.5% to 3.8%) (Table 3). Considering an overall mean incidence rate of 5.77/1,000 person-years, incidence increased from 4.59/1,000 person-years in 1992 to 7.25/1,000 person-years in 2014. This estimate was based on the best-fitting model, adjusting for age and sex and with a random intercept and slope term for GPRN. Despite their relatively low numbers of person-years, the highest age groups contributed most to the positive trend, showing the strongest increase in dementia incidence over time. Between the GPRNs we found variation in the trend, with estimated standard deviations of 0.38 and 0.02 for the random intercept and slope terms, respectively. Table 4 shows the variation: the incidence rate ratio per year indicates the individual slope per GPRN, as fitted through model 1. The estimate for the negative binomial dispersion parameter was 31.73 (standard error 4.92), indicating substantial extra variation in the counts. Furthermore, there were no differential trends according to age and sex, as can be seen from the analysis of interaction terms (models 3 and 4; S1 Table). However, independent of time, the incidence rate for females was estimated to be 1.08 (95% CI 1.04 to 1.13) times higher than for males. Also independent of time, rates increased with age, approximately doubling with each 5-y increment in age, with a slower increase towards older age groups (Table 3).

**Table 3:** Results of negative binomial regression analysis, giving incidence rate ratios of change in dementia incidence.

Analysis	Slope (95% CI)	Standard error	*p*-Value	Rate ratio (95% CI)
**Model 1**				
Year^1^	0.021	0.008	0.011	1.021 (1.005 to 1.038)
Age 65–69^2^	0.981	0.064	<0.001	2.667 (2.352 to 3.027)
Age 70–74^2^	2.016	0.059	<0.001	7.508 (6.686 to 8.433)
Age 75–79^2^	2.947	0.057	<0.001	19.049 (17.043 to 21.285)
Age 80–84^2^	3.612	0.056	<0.001	37.040 (33.191 to 41.343)
Age 85+^2^	4.040	0.056	<0.001	56.826 (50.962 to 63.407)
Female^3^	0.078	0.022	<0.001	1.081 (1.035 to 1.129)
**Model 2**				
Change of trend in years ≥ 2003 compared to trend in years < 2003	−0.025 (–0.062 to 0.011)	0.0186	0.171	

Both models adjusted for age (5-y age groups), sex, and random intercept and slope for general practitioner registration network. Model 2 included a piecewise linear spline allowing one knot in the year 2003.

^1^Estimations relative to the previous calendar year.

^2^Estimations relative to persons aged 60–64 y.

^3^Estimations relative to males.

**Table 4:** Variation in trends between general practitioner registration networks as estimated using model 1.

GPRN	Random slope (SD 0.022)	Incidence rate ratio per year
RNUH-LEO	−0.033	0.988
SMILE	−0.022	0.999
CMR	−0.009	1.012
RNH	−0.004	1.017
Trans	−0.003	1.019
NIVEL-PCD	0.004	1.026
IPCI	0.015	1.037
JHN	0.016	1.038
HAG	0.036	1.058

“Random slope” indicates the deviation from the fitted slope for calendar year in model 1 (0.021).

GPRN, general practitioner registration network; SD, standard deviation.

In the piecewise linear spline model, the trend over the years from 2003 to 2014 showed a small, nonsignificant change compared to the trend over the years prior to 2003 (−0.025; 95% CI −0.062 to 0.011) (Table 3). Thus, there was no significant change in the trend of recorded dementia incidence rate since the Dutch national dementia care program was launched in 2003.

When taking into account all available years, including the years between 1986 and 1992 with fewer than 10,000 person-years, findings did not notably change (incidence rate ratio 1.022; 95% CI 1.006 to 1.039) (model 5, S1 Table). Also, when we used Poisson regression instead of negative binomial regression, we observed a comparable increase in dementia incidence over time (model 6, S1 Table).

## Discussion

This study evaluated whether there was a declining trend in dementia incidence rate in the Netherlands, using a real-world sample of routinely collected data from primary care networks comprising over 800,000 people aged 60 y and over. Pooled data from nine GPRNs showed a dementia incidence rate ratio of 1.021 (95% CI 1.005 to 1.038) per year between 1992 and 2014, with higher incidence rates among women than among men and no significant change since the start of a national dementia program in 2003.

This study is unique in that it combines data from virtually all of the GPRNs of one country. Strengths are the relatively long period of observation (23 y) and the large numbers of observed person-years at risk (over 4 million) and incident dementia cases (over 23,000). Other strengths are the representativeness of the studied population: nearly all Dutch inhabitants are registered with one general practice, and the included GPRNs cover inhabitants from all geographical areas [14]; thus, there was no selection or attrition bias in this dynamic cohort. A limitation may be the potential underestimation of dementia diagnoses, especially for mild dementia [22,23]. Although diagnostic criteria for dementia have not substantially changed over the last decades, in early phases of the disease the diagnosis is often not formally made by GPs, even if suspected [24]. However, this leads to high specificity, and therefore high internal validity of diagnostic labels by the GPs [25]. Despite a potentially low sensitivity, the long period of observation ensures that patients with moderate to severe dementia are likely to eventually receive a diagnostic label in their EHR. Nevertheless, patient and GP awareness of dementia and individual GPs’ perspectives of disease may have changed and thus inflated recorded incidence rates over time. For example, in 2003 a national dementia care program was launched [17], followed by programs to finance and facilitate integrated dementia care [26,27], which may have supported both diagnosis and care in primary care. However, our analyses did not show any change in the overall incidence rate trend following initiation of these programs compared to the years prior to their introduction, nor did including a longer time period affect the overall incidence rate trend. Nevertheless, secular trends towards diagnosis in earlier stages of dementia are suggested by studies that compared clinical diagnosis with Mini-Mental State Examination (MMSE) scores over time, and found higher scores on the MMSE in patients diagnosed with dementia in more recent years [11,28]. Another limitation might be the inability to fully correct for increased overall life expectancy over time and the national development towards an increasing share of non-institutionalized older people [29]. Since people living in nursing homes are not registered with a GP, this may have contributed to increased numbers of dementia diagnoses in GP registries. Although we corrected for age in 5-y groups, these phenomena could have affected the incidence rates in the highest age group, which lacked an upper age limit (85 y and over). However, a differing trend by age was not confirmed by an analysis allowing different time trends across age groups (model 3, S1 Table). We cannot exclude the possibility that the trend towards a small increase in dementia incidence rate that was found in our study reflects a balance of increased awareness, earlier diagnosis, and an increasing percentage of community-dwelling older people on the one hand and stable or even declining dementia incidence rates on the other hand. Another limitation of the study might be the difference in available data between GPRNs to calculate dementia rates. Although calculation methods did not differ essentially within GPRNs, differences in establishing incident cases (using episode lists in some and problem lists in others) and defining the denominator (using the number of person-years at risk or the number of registered persons at risk) might explain some of the variation in morbidity estimates across the studied GPRNs. Nevertheless, additional analyses comparing trends within one GPRN that provided both the number of person-years and the number of persons per year showed no differences in trend between the two methods (estimates differed only from the fourth decimal; S2 Table). Also, previous analyses of morbidity data from all Dutch GPRNs showed that neither population nor practice characteristics could explain the variation in incidence and prevalence estimates between practices or GPRNs [14,30]. Finally, a disadvantage of studying incidence rates is that this approach cannot directly be used for future projections of dementia prevalence, since this also requires integration with dementia-specific mortality rates. On the other hand, age-specific incidence rates are not influenced by demographic ageing and show less variation than prevalence rates within GPRNs [14].

The dementia incidence rates reported here are similar to those found in a literature review on rates in Europe and the US that reported estimates of 7.1 to 19.2 and 12.8 to 36.2 per 1,000 person-years for people aged 75–79 and 80–84 y, respectively [31]. Different trends have been reported for men and women, though results are conflicting [11,32,33]. We found similar trends for both sexes, even though overall rates were higher among women. So far, few studies have presented incidence data using consistent research methods across multiple time points [12]. The Rotterdam Study reported a nonsignificant decline in dementia occurrence between 1990 and 2005 [9]. A recent study in the UK found a decline in incidence between 1989 and 2011 that was significant among men [33]. In the same cohort, a significant 22% decrease in prevalence was found [5]. In the Framingham Heart Study, four epochs between 1977 and 2008 showed a decline in the incidence of dementia, especially for vascular dementia and in those who had a high school diploma. The decline in incidence rate was mainly seen between the first two epochs, while rates stabilized from the 1990s onwards, suggesting that the overall decline was driven mainly by data from the years prior to this period [10]. Compared to our findings, age-specific rates were similar, and considering the time period since the 1990s, only small changes in the rates over time were found in both studies. Other studies in Sweden, France, and the US found small, nonsignificant changes in incidence rates over time [7,8,11,34]. Based on integration of prevalence and mortality figures, a study conducted in Stockholm, Sweden, suggested the possibility of reduced dementia incidence [35], and findings from a study in Zaragoza, Spain, showed a significant reduction in dementia prevalence only in men [32]. Main limitations of these studies concerned decreasing response rates [5,11,32], with varying ability to assess the potential effects of such changes on the findings. Inflation of estimates may have taken place if nonstandardized diagnostic criteria were used [7,34] or when medical records were retrospectively used to supplement incomplete information [9,32], because of the increased inclusiveness of broader diagnostic criteria across time [5,32].

The overall increase in diagnosis of dementia of 2.1% in general practice registries reported here differs from the declining dementia incidence rate in some population-based studies. These studies were specifically designed to measure dementia incidence in fixed cohorts, rather than in dynamic populations such as the ones reported here. At the same time, our findings do not preclude the possibility that age-specific prevalence rates are stabilizing, depending on dementia-specific mortality rates [13]. Although improved vascular risk management has been linked to the alleged decrease in dementia incidence in previous studies, favorable trends with respect to smoking and hypertension may have been reversed by increasing rates of obesity and type 2 diabetes mellitus [10]. Perhaps the gains from improved cardiovascular prevention were capitalized in the 1970s and 1980s, yielding relatively stable trends over the last decades. The complex interplay between these and other factors, like survival after cardiovascular disease, will require further study to determine their net effect on dementia occurrence. Irrespective of the question of to what extent the figures presented here exactly reflect incidence rates of dementia in a Western population, our data indicate that the burden of work for physicians and nurses in general practice associated with newly diagnosed dementia has not declined in the past two decades, although there may have been a shift to milder spectrum disorder. With an ever increasing older population, the absolute capacity required for the care of dementia patients in general practice can still be expected to double every 20 y, despite observed decreasing dementia incidence rates in some specific populations, especially before the 1990s.

Results from other population registries or public health records in high-income countries are needed to confirm our findings, and to study demographics and the impact of dementia risk factors on incidence trends in ageing societies. Direct comparison of such registries with epidemiologic studies performed simultaneously in the same area may help to explain the apparent discrepancy between the current findings and those in specific cohort populations. In this study on longitudinal, real-world primary care data, we have found a small absolute increase in dementia incidence rates over the last two decades. Although this finding appears to be in contrast with recent reports of attenuating incidence rates and dementia occurrence, the exact reasons remain to be explored, highlighting the need for greater understanding of complex time trends in dementia incidence.

## Supporting information

S1 Table Results of regression analysis, giving incidence rate ratios of change in dementia incidence. (DOC)

S2 Table Results of analyses with registered person-years and registered persons in one general practitioner registration network (Continuous Morbidity Registration Nijmegen). (DOC)

S1 Text RECORD checklist. (DOCX)

## Abbreviations

EHR: electronic health record
GP: general practitioner
GPRN: general practice registration network
ICPC: International Classification of Primary Care
