# Supplementary material for: Dementia incidence trend over 1992-2014 in the Netherlands: Analysis of primary care data
Source: PLoS Med. 2017 Mar 7;14(3):e1002235. doi: 10.1371/journal.pmed.1002235 (PMC5340347; doi:10.1371/journal.pmed.1002235)
Supplement: S1 Table — (DOC) [file pmed.1002235.s001.doc]

| **S1 Table. Results of regression analysis giving incidence rate ratios of change in dementia incidence** | | | | |
| --- | --- | --- | --- | --- |
|  | Regression coefficient | Standard Error | *p*-Value | Rate ratio (95% CI) |
| Model 2  Change of trend in years ≥2003 compared to trend in years <2003  Years <2003  Years ≥2003 | -0.025  (-0.062 to 0.011)  0.040  0.015 | 0.0186  0.0153  0.0111 | 0.171  0.009  0.189 | 1.041 (1.010 to 1.073)  1.015 (-0.007 to 0.036) |
| Model 3 | 0.03072  -0.01252  -0.01533  -0.01034  -0.00809  -0.00779 | 0.01461  0.01470  0.01356  0.01315  0.01306  0.01302 | 0.036  0.39  0.26  0.43  0.56  0.55 | 1.031 (1.002 to 1.061) |
| Year Interaction terms for age  Age 65-69  Age 70-74  Age 75-79  Age 80-84  Age 85+ |
| Model 4 | 0.01953  0.00278 | 0.00880  0.00471 | 0.026  0.555 | 1.020 (1.002 to 1.037) |
| Year  Interaction term for sex  Female |
| Model 5  Year | 0.02212 | 0.00809 | 0.00628 | 1.022 (1.006 to 1.039) |
| Model 6  Year | 0.01763 | 0.00762 | 0.021 | 1.018 (1.003 to 1.033) |
| **Model 2**: negative binomial regression analysis with random intercept and slope for GPRN, adjusted for age and sex, including piecewise linear spline allowing one knot in the year 2003  **Model 3**: negative binomial regression analysis with random intercept and slope for GPRN, adjusted for age and sex with an interaction term for age  **Model 4:** negative binomial regression analysis with random intercept and slope for GPRN, adjusted for age and sex with an interaction term for sex  **Model 5:** negative binomial regression analysis with random intercept and slope for GPRN, adjusted for age and sex, including all available years (1986-2014)  **Model 6:** Poisson regression analysis with random intercept and slope for GPRN, adjusted for age and sex | | | | |
