# Supplementary material for: Dementia incidence trend over 1992-2014 in the Netherlands: Analysis of primary care data
Source: PLoS Med. 2017 Mar 7;14(3):e1002235. doi: 10.1371/journal.pmed.1002235 (PMC5340347; doi:10.1371/journal.pmed.1002235)
Supplement: S2 Table — (DOC) [file pmed.1002235.s002.doc]

| **S2 Table. Results of analyses with registered person-years and registered persons in one general practitioner registration network ( Continuous Morbidity Registration Nijmegen)** | | | | |
| --- | --- | --- | --- | --- |
|  | Regression coefficient for ‘year’ | Standard Error | *p*-Value | Rate ratio (95% CI) |
| **Number of person years** | 0.01151 | 0.00921 | 0.212 | 1.012 (0.993 – 1.030) |
| **Number of registered persons** | 0.01187 | 0.00915 | 0.195 | 1.012 (0.994 – 1.030) |
