# Supplementary material for: Dementia incidence trend over 1992-2014 in the Netherlands: Analysis of primary care data
Source: PLoS Med. 2017 Mar 7;14(3):e1002235. doi: 10.1371/journal.pmed.1002235 (PMC5340347; doi:10.1371/journal.pmed.1002235)
Supplement: S1 Text — (DOCX) [file pmed.1002235.s003.docx]

**S1 Text: RECORD Checklist for the manuscript entitled: “Dementia incidence trend over 1992-2014 in the Netherlands: analysis of primary care data”**

**The RECORD statement – checklist of items, extended from the STROBE statement, that should be reported in observational studies using routinely collected health data.**

|  | **Item No.** | **STROBE items** | **Location in manuscript where items are reported** | **RECORD items** | **Location in manuscript where items are reported** |
| --- | --- | --- | --- | --- | --- |
| **Title and abstract** | | | | | |
|  | 1 | (a) Indicate the study’s design with a commonly used term in the title or the abstract (b) Provide in the abstract an informative and balanced summary of what was done and what was found | a) See title: “Dementia incidence trend over 1992-2014: analysis of primary care data in the Netherlands” [p1]  b) Reply: Done [p2] | RECORD 1.1: The type of data used should be specified in the title or abstract. When possible, the name of the databases used should be included.  RECORD 1.2: If applicable, the geographic region and timeframe within which the study took place should be reported in the title or abstract.  RECORD 1.3: If linkage between databases was conducted for the study, this should be clearly stated in the title or abstract. | 1.1) Type of data is specified in title and abstract. [p1-2] Due to the number of databases it is not possible to name them all in the abstract.  1.2) Done [p1-2]  1.3) No linkage was conducted. |
| **Introduction** | | | | | |
| Background rationale | 2 | Explain the scientific background and rationale for the investigation being reported | Done [p4] | | |
| Objectives | 3 | State specific objectives, including any prespecified hypotheses | Done [p5-6] | | |
| **Methods** | | | | | |
| Study Design | 4 | Present key elements of study design early in the paper | In title and abstract an overview of the study design is given. Further description follows in the method section first paragraph: “For this study, all eleven GPRNs that routinely and continuously collected data on morbidity and mortality in the Netherlands over the last decades were invited to participate.” and the third paragraph: “For all databases, count data of incident cases and person-years at risk per year, sex and age group were directly obtained or calculated from the anonymized data.” [p7] | | |
| Setting | 5 | Describe the setting, locations, and relevant dates, including periods of recruitment, exposure, follow-up, and data collection | See methods section, first paragraph: “GPRNs collect and manage EHR data of large numbers of associated GP practices. Most operate regionally, some nationally”, and third paragraph: “Data of all available years with at least 10,000 observed person-years were used for data analysis, in order to avoid unrepresentative sample years, resulting in data for the years 1992 through 2014.” [p7-8] | | |
| Participants | 6 | *(a) Cohort study* - Give the eligibility criteria, and the sources and methods of selection of participants. Describe methods of follow-up  *Case-control study* - Give the eligibility criteria, and the sources and methods of case ascertainment and control selection. Give the rationale for the choice of cases and controls  *Cross-sectional study* - Give the eligibility criteria, and the sources and methods of selection of participants  *(b) Cohort study* - For matched studies, give matching criteria and number of exposed and unexposed  *Case-control study* - For matched studies, give matching criteria and the number of controls per case | a) See methods section, first paragraph: “Databases of GPRNs were considered eligible if data were available for at least five years and registration or extraction methods had not substantially changed over time (table 1).”, and third paragraph: “data on all people aged 60 and over were used.” [p7-8] Methods of follow up: Not applicable.  b) Not applicable. | RECORD 6.1: The methods of study population selection (such as codes or algorithms used to identify subjects) should be listed in detail. If this is not possible, an explanation should be provided.  RECORD 6.2: Any validation studies of the codes or algorithms used to select the population should be referenced. If validation was conducted for this study and not published elsewhere, detailed methods and results should be provided.  RECORD 6.3: If the study involved linkage of databases, consider use of a flow diagram or other graphical display to demonstrate the data linkage process, including the number of individuals with linked data at each stage. | 6.1) This information is provided in the methods section (Paragraph named data extraction): “For all databases, count data of incident cases and person-years at risk per year, sex and age group were directly obtained or calculated from the anonymized data.” [p8]  6.2) Not applicable  6.3) Not applicable |
| Variables | 7 | Clearly define all outcomes, exposures, predictors, potential confounders, and effect modifiers. Give diagnostic criteria, if applicable. | This information is provided in the methods section (Paragraph named: “Data extraction”) [p8] | RECORD 7.1: A complete list of codes and algorithms used to classify exposures, outcomes, confounders, and effect modifiers should be provided. If these cannot be reported, an explanation should be provided. | 7.1) Only one code was used (ICPC P70), considerations regarding other codes are provided in the methods section (Paragraph named: “Data extraction”) [p8] |
| Data sources/ measurement | 8 | For each variable of interest, give sources of data and details of methods of assessment (measurement).  Describe comparability of assessment methods if there is more than one group | This information is provided in the methods section (Paragraph named: “Data extraction”) [p8] | | |
| Bias | 9 | Describe any efforts to address potential sources of bias | This information is provided in the methods section (Paragraph named: “Statistical analysis”) [p9] | | |
| Study size | 10 | Explain how the study size was arrived at | See method section, first paragraph: “For this study, all eleven GPRNs that routinely and continuously collected data on morbidity and mortality in the Netherlands over the last decades were invited to participate.” [p7] | | |
| Quantitative variables | 11 | Explain how quantitative variables were handled in the analyses. If applicable, describe which groupings were chosen, and why | Not applicable | | |
| Statistical methods | 12 | (a) Describe all statistical methods, including those used to control for confounding  (b) Describe any methods used to examine subgroups and interactions  (c) Explain how missing data were addressed  (d) *Cohort study* - If applicable, explain how loss to follow-up was addressed  *Case-control study* - If applicable, explain how matching of cases and controls was addressed  *Cross-sectional study* - If applicable, describe analytical methods taking account of sampling strategy  (e) Describe any sensitivity analyses | a) This information is provided in the methods section (Paragraph named: “Statistical analysis”) [p9]  b) Reply: This information is provided in the methods section (Paragraph named: “Statistical analysis”) [p9]  c) not applicable.  d) not applicable.  e) This information is provided in the methods section (Paragraph named: “statistical analysis”): “Furthermore, to test the hypothesis that the recorded dementia incidence increased as a result of increased awareness and case manager-led integrated dementia care, a piecewise linear spline was included in the model, with an internal knot at year 2003 (when the National Dementia care Program was launched in the Netherlands).] Additional sensitivity analyses were performed using Poisson regression instead of Negative Binomial regression, and including all available data (1986-2014) in the Negative Binomial regression.” [p9] | | |
| Data access and cleaning methods |  | .. |  | RECORD 12.1: Authors should describe the extent to which the investigators had access to the database population used to create the study population.  RECORD 12.2: Authors should provide information on the data cleaning methods used in the study. | This information is provided in the methods section (paragraph named ‘data extraction’) [p8]:  12.1) “For all databases, count data of incident cases and person-years at risk per year, sex and age group were directly obtained or calculated from the anonymized data.”  12.2) “At the start of each calendar year, prevalent dementia cases were excluded from both the numerator and denominator.” |
| Linkage |  | .. |  | RECORD 12.3: State whether the study included person-level, institutional-level, or other data linkage across two or more databases. The methods of linkage and methods of linkage quality evaluation should be provided. | 12.3) No linkage was conducted for this study. |
| **Results** | | | | | |
| Participants | 13 | (a) Report the numbers of individuals at each stage of the study (*e.g.*, numbers potentially eligible, examined for eligibility, confirmed eligible, included in the study, completing follow-up, and analysed)  (b) Give reasons for non-participation at each stage.  (c) Consider use of a flow diagram | a) This information is provided in the results section, first two paragraphs and Table 1 and Fig. 1. [p10-11]  b) Reply: non-participation of 2 GPRNs is provided in the first paragraph of the result section. [p10]  c) Reply: Not applicable | RECORD 13.1: Describe in detail the selection of the persons included in the study (*i.e.,* study population selection) including filtering based on data quality, data availability and linkage. The selection of included persons can be described in the text and/or by means of the study flow diagram. | 13.1) This information is provided in the methods section (Paragraph named: “data extraction”): “for each calendar year, data on all people aged 60 and over were used.” |
| Descriptive data | 14 | (a) Give characteristics of study participants (*e.g.*, demographic, clinical, social) and information on exposures and potential confounders  (b) Indicate the number of participants with missing data for each variable of interest  (c) *Cohort study* - summarise follow-up time (*e.g.*, average and total amount) | a) Not applicable  b) Not applicable  c) Not applicable | | |
| Outcome data | 15 | *Cohort study* - Report numbers of outcome events or summary measures over time  *Case-control study* - Report numbers in each exposure category, or summary measures of exposure  *Cross-sectional study* - Report numbers of outcome events or summary measures | See Fig. 1; number of events and person-years per year. | | |
| Main results | 16 | (a) Give unadjusted estimates and, if applicable, confounder-adjusted estimates and their precision (e.g., 95% confidence interval). Make clear which confounders were adjusted for and why they were included  (b) Report category boundaries when continuous variables were categorized  (c) If relevant, consider translating estimates of relative risk into absolute risk for a meaningful time period | a) This information is provided in the results section. All results are adjusted for age, sex, and GPRN, since this gave the best model fit. The supplementary appendix provides additional analyses. “We incorporated GPRN random intercept and slope terms into the Negative Binomial regression models to allow for GPRN specific trends of dementia over time. The most parsimonious random structure was chosen based on the likelihood ratio test. The time-rate relation was adjusted for age (in 5 year age groups) and sex. We also investigated whether the time-rate relation differed across age groups and sex by adding the appropriate interaction terms to the model.”  b) This is applicable only for age. Methods section: “The time-rate relation was adjusted for age (in 5 year age groups) and sex”  c) See result section: “Considering an overall mean incidence rate of 5.77/1000 person-years, incidence increased from 4.59 in 1992 to 7.25/1000 person-years in 2014.” [p12] | | |
| Other analyses | 17 | Report other analyses done—e.g., analyses of subgroups and interactions, and sensitivity analyses | This information is provided in the result section, last two paragraphs , and in the supplementary appendix. [p12-13] | | |
| **Discussion** | | | | | |
| Key results | 18 | Summarise key results with reference to study objectives | See discussion section, first paragraph: “This study evaluated whether there was a declining trends in dementia incidence rate in the Netherlands, using a real-world sample of routinely collected data from primary care networks comprising over 800,000 people aged 60 and over. Pooled data from nine GPRNs showed a dementia incidence rate ratio of 1.021 (95%CI 1.005 to 1.038) per year between 1992 and 2014, with higher incidence rates among women compared to men and no significant change since the start of a national dementia program in 2003.” [p15] | | |
| Limitations | 19 | Discuss limitations of the study, taking into account sources of potential bias or imprecision. Discuss both direction and magnitude of any potential bias | This information is provided in the discussion section, second paragraph. [p15-17] | RECORD 19.1: Discuss the implications of using data that were not created or collected to answer the specific research question(s). Include discussion of misclassification bias, unmeasured confounding, missing data, and changing eligibility over time, as they pertain to the study being reported. | 19.1) This information is provided in the discussion section, second paragraph. [p14-16] |
| Interpretation | 20 | Give a cautious overall interpretation of results considering objectives, limitations, multiplicity of analyses, results from similar studies, and other relevant evidence | This information is provided in the discussion section, second-to-last paragraph. [p18-19] | | |
| Generalisability | 21 | Discuss the generalisability (external validity) of the study results | See discussion section, second and last paragraph: “Therefore, as for the trend towards a small increase that was found in our study, we cannot exclude that it reflects a balance of increased awareness, earlier diagnosis and an increasing percentage of community dwelling older people on the one hand and stable or even declining dementia incidence rates on the other hand. [p16]” and “. Although this finding appears to be in contrast with recent reports of attenuating incidence rates and dementia occurrence the exact reasons remain to be explored and highlight the need for greater understanding of complex time-trends in dementia-incidence.” [p19] | | |
| **Other Information** | | | | | |
| Funding | 22 | Give the source of funding and the role of the funders for the present study and, if applicable, for the original study on which the present article is based | This information is provided in the designated boxes on the submission platform as requested by the editorial office. | | |
| Accessibility of protocol, raw data, and programming code |  |  |  | RECORD 22.1: Authors should provide information on how to access any supplemental information such as the study protocol, raw data, or programming code. | 22.1) This information is provided in the methods section (paragraph ‘data usage and data availability’): To access the data, permission is needed from the individual GPRNs that own the data. The data of this study is stored, and can be requested from the AMC, department of General Practice ([huisartsgeneeskunde@amc.uva.nl](mailto:huisartsgeneeskunde@amc.uva.nl)). The programming codes can be requested from the corresponding author.” [p10] |

*Reference: Benchimol EI, Smeeth L, Guttmann A, Harron K, Moher D, Petersen I, Sørensen HT, von Elm E, Langan SM, the RECORD Working Committee. The REporting of studies Conducted using Observational Routinely-collected health Data (RECORD) Statement. *PLoS Medicine* 2015; in press.

*Checklist is protected under Creative Commons Attribution ([CC BY](http://creativecommons.org/licenses/by/4.0/)) license.
